# Safety and efficacy study of laparoscopic or robotic radical surgery using an endoscopic stapler for inhibiting tumour spillage of cervical malignant neoplasms evaluating survival (SOLUTION): a multi-centre, open-label, single-arm, phase II trial protocol

**DOI:** 10.1186/s12885-022-09429-z

**Published:** 2022-03-26

**Authors:** Soo Jin Park, Tae Wook Kong, Taehun Kim, Maria Lee, Chel Hun Choi, Seung-Hyuk Shim, Ga Won Yim, Seungmee Lee, Eun Ji Lee, Myong Cheol Lim, Suk-Joon Chang, Sung Jong Lee, San Hui Lee, Taejong Song, Yoo-Young Lee, Hee Seung Kim, Eun Ji Nam

**Affiliations:** 1 Department of Obstetrics and Gynecology, Seoul National University College of Medicine, 101 Daehak-Ro Jongno-Gu, Seoul, 03080 Republic of Korea; 2 Division of Gynecologic Oncology, Department of Obstetrics and Gynecology, Ajou University School of Medicine, Suwon, 16499 South Korea; 3 Department of Obstetrics and Gynecology, Seoul Metropolitan Government-Seoul National University Boramae Medical Center, Seoul, 07061 South Korea; 4 Department of Obstetrics and Gynecology, Samsung Medical Center, Sungkyunkwan University School of Medicine, Seoul, 06351 Republic of Korea; 5 Department of Obstetrics and Gynecology, Research Institute of Medical Science, Konkuk University School of Medicine, Seoul, 05030 South Korea; 6 Department of Obstetrics and Gynecology, Dongguk University College of Medicine, Goyang, 10326 Republic of Korea; 7 Department of Obstetrics and Gynecology, Keimyung University School of Medicine, Daegu, 42601 Republic of Korea; 8 Division of Tumor Immunology, Research Institute, and Center for Gynecologic Cancer & Center for Clinical Trial, Hospital, and Department of Cancer Control & Population Health, GSCSP, National Cancer Center, Goyang, 10408 South Korea; 9 Department of Obstetrics and Gynecology, Seoul St. Mary’s Hospital, College of Medicine, The Catholic University of Korea, 06591 Seoul, Republic of Korea; 10 Department of Obstetrics and Gynecology, Wonju Severance Christian Hospital, Yonsei University College of Medicine, Wonju, 26426 Republic of Korea; 11 Department of Obstetrics and Gynecology, Kangbuk Samsung Hospital, Sungkyunkwan University School of Medicine, Seoul, 03181 South Korea; 12 Hereditary Cancer Clinic, Cancer Prevention Center, Yonsei Cancer Center, and Department of Obstetrics and Gynecology, Institute of Women’s Life Medical Science, Women’s Cancer Clinic, Yonsei University College of Medicine, Seoul, 03722 Republic of Korea

**Keywords:** Cervical cancer, Minimally invasive surgery, Endoscopic stapler, Recurrence, Survival

## Abstract

**Background:**

The Laparoscopic Approach to Cervical Cancer trial and Surveillance, Epidemiology, and End Results program database study demonstrated that minimally invasive radical hysterectomy was inferior to abdominal radical hysterectomy in terms of disease recurrence and survival. Among risk factors related to poor prognosis after minimally invasive surgery (MIS), tumour spillage during intracorporeal colpotomy became a significant issue. Thus, we designed this trial to evaluate the efficacy and safety of minimally invasive radical hysterectomy using an endoscopic stapler for early-stage cervical cancer.

**Methods:**

This trial is a prospective, multi-centre, open-label, single-arm, non-inferiority phase II study. The nine organisations will participate in this trial after the approval of the institutional review board. Major eligibility criteria include women aged 20 years or older with cervical cancer stage IB1 squamous cell carcinoma, adenocarcinoma, or adenosquamous carcinoma according to the revised 2009 FIGO staging system who will undergo type B2 or C hysterectomy by MIS. The primary endpoint is the 4.5-year disease-free survival (DFS) rate between abdominal radical hysterectomy and MIS using an endoscopic stapler. For calculating the sample size, we hypothesised that the 4.5-year DFS rate after MIS using an endoscopic stapler is assumed to be the same after abdominal radical hysterectomy at 90.9%, and the non-inferiority margin was 7.2%. When we consider a three-year accrual and 4.5-year follow-up, at least 13 events must happen, requiring a total of 111 patients assuming a statistical power of 80% and the one-tailed test of 5% significance. A total of 124 patients is needed, considering a drop-out rate of 10%.

**Discussion:**

We expect intracorporeal colpotomy using an endoscopic stapler may prevent tumour spillage during MIS for stage IB1 cervical cancer, showing a comparable prognosis with abdominal radical surgery.

**Trial registration:**

ClinicalTrials.gov; NCT04370496; registration date, May 2020.

## Background

Cervical cancer is the fourth frequent cancer in women [1], and in case of localized disease, the 5-year survival rate is 92.5%, showing a relatively good prognosis [2]. Treatment of early-stage cervical cancer is based on radical hysterectomy and pelvic lymphadenectomy, and adjuvant concurrent radiotherapy (CCRT) may be performed depending on risk factors. Recently, two paradigm-shifting studies reported that radical hysterectomy using minimally invasive surgery (MIS) might be inferior to abdominal radical hysterectomy for treating early-stage cervical cancer [3, 4]. The Laparoscopic Approach to Cervical Cancer (LACC) trial, a non-inferiority randomized controlled trial aimed to compare disease-free survival at 4.5 years between patients undergoing MIS and abdominal radical hysterectomy, showed a higher risk of disease recurrence in MIS group, leading to its early termination [3]. Similarly, an additional retrospective study from the National Cancer Database of the United States described a higher risk of all-cause mortality in MIS group rather than open surgery group [4]. An additional retrospective study from the National Cancer Database of the United States also demonstrated that MIS group showed a higher risk of all-cause mortality than open surgery group [4]. Thereafter, major organisations revised their guidelines for surgical management of early-stage cervical cancer, favouring abdominal radical hysterectomy instead of MIS [5, 6].

Among risk factors to increase disease recurrence and reduce the prognosis after MIS, many experts suggest the following four factors. First, intracorporeal colpotomy, defined as intra-abdominal resection of the vagina, can spill tumour cells into the peritoneal cavity [3]. Second, a uterine manipulator inserted into the endometrium can lead to tumour spillage via fallopian tubes [7]. Third, capnoperitoneum during MIS can stimulate and proliferate tumour cells spilt into the peritoneal cavity [3]. Fourth, tumour cells spilt into the pelvic cavity can move to the upper abdominal cavity by Trendelenburg position during MIS [8].

Above all, tumour spillage during MIS is a major issue related to disease recurrence and survival in various types of cancer surgery. In early-stage ovarian cancer, intraoperative rupture accounts for upstaging, which requires adjuvant chemotherapy [9]. On the other hand, no difference in survival between MIS and open surgery has been reported in the bladder and colorectal cancer [10, 11]. However, it should be noted that tumour cells only spread within the lumen because tumours are often encapsulated in the bladder and bowel lumen, and then they are little exposed into the peritoneal cavity [10, 11].

To prevent tumour spillage during minimally invasive radical hysterectomy, extracorporeal colpotomy and transvaginal closure of the vaginal stump have been tried in several studies [12, 13]. However, this surgical technique using the vaginal approach is not easy for postmenopausal patients with the atrophic vagina because the vaginal cavity is narrow, and it is not feasible for some surgeons who are not proficient in vaginal surgery.

This phase II study aims to evaluate 4.5-year disease-free survival rate of minimally invasive radical hysterectomy implementing the surgical technique using an endoscopic stapler. During this procedure, tubal ligation and sealing of cervical tumours by resectioning the vagina using an endoscopic stapler are expected to prevent tumour spillage into the peritoneal cavity.

## Methods/design

### Trial design

SOLUTION (Safety Of Laparoscopic or robotic radical surgery Using an endoscopic sTapler for Inhibiting tumOr spillage of cervical Neoplasms) trial is a prospective, multi-centre, open-label, single-arm, non-inferiority phase II study recruited from ten gynecologic cancer centres in the Republic of Korea. Each of the nine organisations has received institutional review board approval in advance, and a single organisation is in the process of approval. Participating organisations are as follows: Seoul National University Hospital; Ajou University Hospital; Seoul Metropolitan Government-Seoul National University Boramae Medical Center; Samsung Medical Center; Konkuk Universit Hospital; Seoul St. Mary’s Hospital; Wonju Severance Christian Hospital; Kangbuk Samsung Hospital; Severance Medical Center and; National Cancer Center (in progress for approval).

### Surgical procedure

**Fig. 1:**
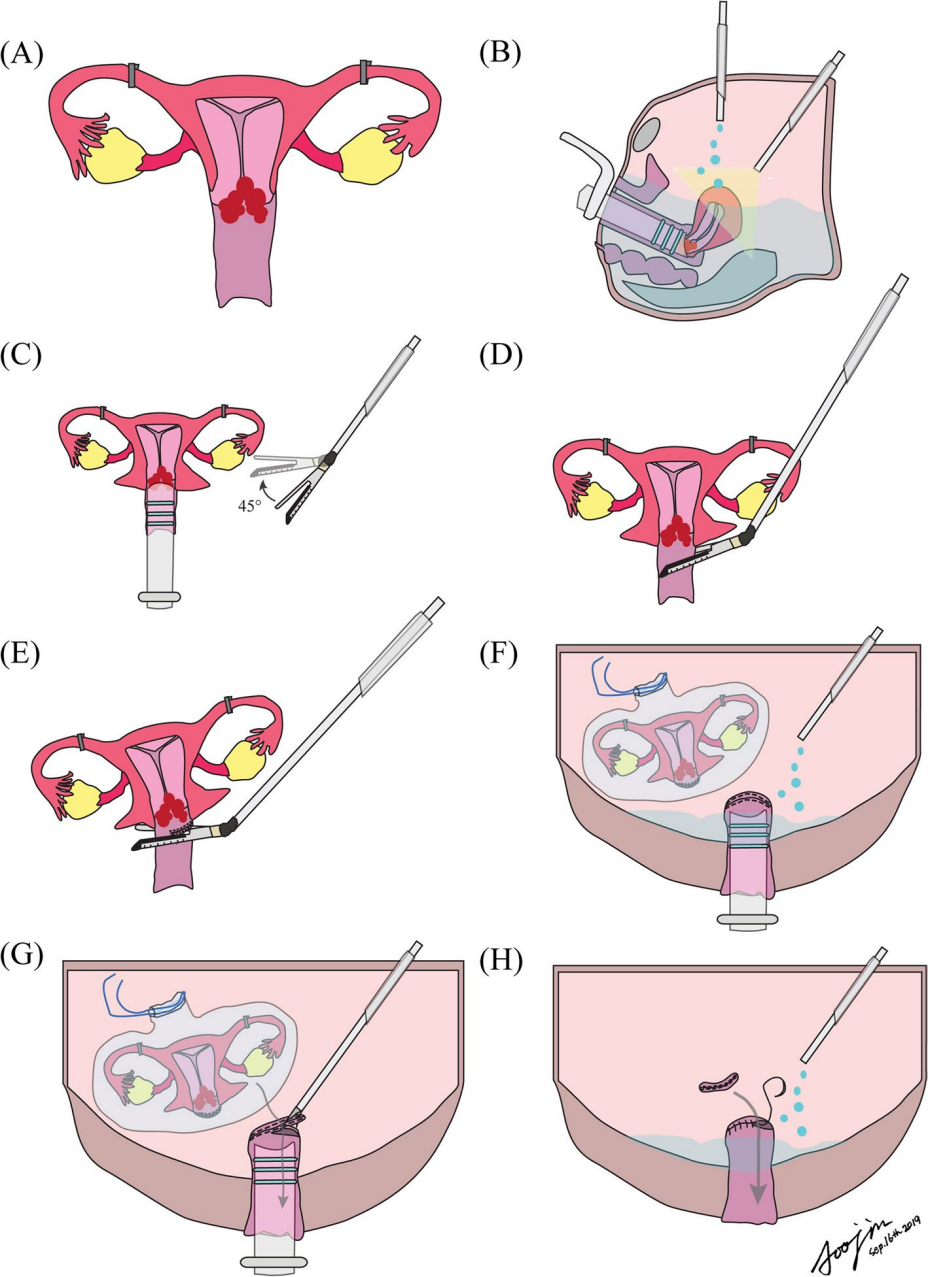
Step-by-step surgical procedure for SOLUTION trial: (**A**) bilateral tubal ligation; (**B**) the first peritoneal washing cytology; (**C**) Flexion of the endoscopic stapler to 45 degrees; (**D**) the first stapling of the vagina; (**E**) the second stapling of the vagina; (**F**) putting the specimen in the endobag, and the second peritoneal washing cytology; (**G**) resection of the stapled vaginal stump and removal of the specimen through the vaginal opening; (**H**) the third peritoneal washing cytology

All patients undergo a laparoscopic or robotic radical hysterectomy in this trial after informed consent based on the surgical technique recommended by the Trial Monitoring Committee of SOLUTION trial [14]. First, after entering the peritoneal cavity, the first peritoneal washing cytology is taken if tumour cells are already present in the peritoneal cavity, tubal ligation is performed with an endoscopic clip to prevent tumour spillage via fallopian tubes, and then a uterine manipulator is inserted. Thereafter, pelvic or para-aortic lymphadenectomy is conducted either before or after radical hysterectomy. In general, we perform complete pelvic lymphadenectomy composed of external, internal and obturator region (level 1) and common iliac including presacral (level 2). However, sentinel lymph node mapping will be allowed at the surgeon’s discretion. Complete pelvic lymphadenectomy will be performed even if sentinel lymph node mapping is performed, which continues regardless of identification of lymph node metastasis on the frozen section. Para-aortic lymphadenectomy is conducted depending on the decision of the operator. After we resect bilateral vesicouterine ligaments, paracervices, uterosacral ligaments, and paracolpiums, the endoscopic stapler (ECHELON FLEX™ Powered Plus Stapler and ECHELON ENDOPATH™ Black reload with open staple height of 4.2 mm and closed staple height of 2.3 mm, South Korea, Ethicon Korea, Johnson and Johnson) are inserted via a 12 mm sized trocar and flexed to a 45-degree angle. Thereafter, we conduct intracorporeal colpotomy by resecting the vagina using the endoscopic stapler two or three times, depending on the width of the vagina. After the uterus is placed in the bag, the second peritoneal washing cytology is performed to evaluate if tumour cells are present in the peritoneal cavity during MIS. Finally, the stapled vaginal stump is resected to retrieve the specimen via the vaginal opening using a monopolar scissor after the vagina is washed several times with clean water. Then, the vaginal stump is endoscopically closed with an absorbable suture (barbed suture or vicryl) after removing the specimen. The third peritoneal washing cytology is taken to evaluate if tumour cells are present in the peritoneal cavity after removal of the specimen (Fig. 1).

**Fig. 2:**
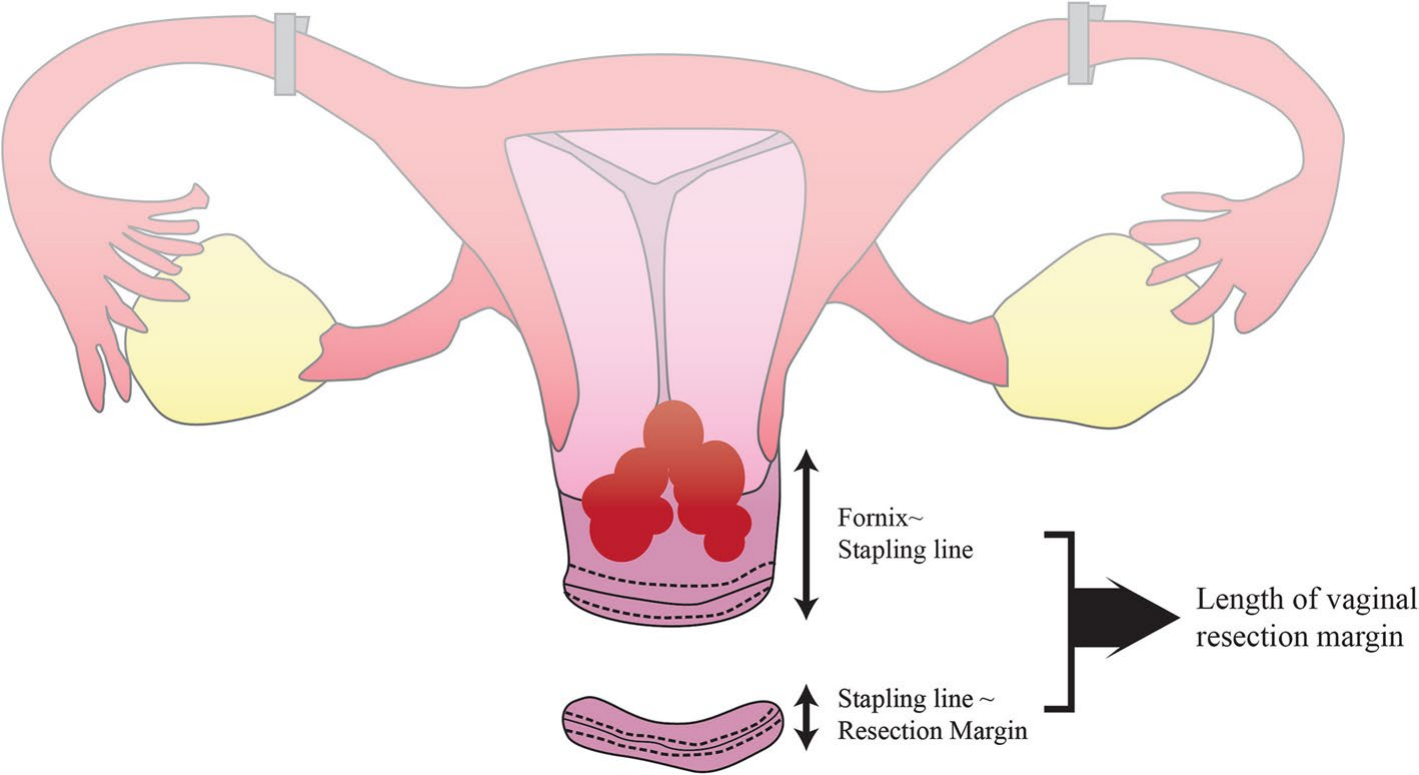
The length between the fornix and the stapled vaginal line and that between the stapled vaginal line and the resected vaginal margin will be measured on the postoperative specimen

After we complete the surgery, we investigate whether the vaginal stapling is complete on the retrieved specimen. Furthermore, we open the stapled margin and then evaluate whether the length of the resected vagina is sufficient by estimating the length between the cervix and the stapled vaginal margin and the length between the stapled vaginal margin and the resected vaginal margin (Fig. 2).

### Surgical quality assurance

The surgeon of each institution participating in this study must fill out an accreditation form to ensure the quality of the surgery and submit at least one unedited surgical video and operation record of type B2 or C radical hysterectomy (Table 1). The Trial Monitoring Committee (TMC) will review the unedited video and operation record for evaluating whether the surgeon properly performs laparoscopic or robotic radical hysterectomy using the endoscopic stapler according to the surgical practice guideline of SOLUTION trial.

**Table 1:** Trial Monitoring Committee Surgical accreditation criteria

Number	Description of Criteria
1	The participating surgeon is a qualified gynecologic oncologists accredited by the Korean Society of Gynecologic Oncology
2	The participating surgeon performs a minimum of 10 documented major oncologic surgeries (cervix, endometrial, ovarian, or vulvar cancer) as the main surgeon per year.
3	The participating surgeon performs a minimum of 10 documented laparoscopic/robotic type B2 or C radical hysterectomy cases as the main surgeon per year.
4	The participating surgeon has attended the endoscopic surgical stapling device seminar with the hands-on workshop.
5	The participating surgeon has performed a minimum of 10 documented successful use of laparoscopic surgical stapling device as the main surgeon.
6	The participating surgeon submitted at least 1 unedited surgical video and the relevant operation record for approval by the TMC - Surgical technique and tissue handling - Competency in identification of proper anatomical structures and adequate dissection of pelvic spaces - Surgical technique with respect to blood loss and prevention of intraoperative injury - Appropriate and timely decision making based on intraoperative findings - Appropriate use and selection of instrumentation for all parts of the procedure

### Participants

Patients satisfying the following inclusion and exclusion criteria will be enrolled in the study.

1. Inclusion criteria
  ① Women aged 20 years or older.
  ② Histologically confirmed squamous cell carcinoma, adenocarcinoma, or adenosquamous carcinoma of the uterine cervix.
  ③ Patients with stage IB1 disease based on the revised 2009 International Federation of Gynecology and Obstetrics (FIGO) staging system.
  ④ Patients undergoing either type B2 or C hysterectomy by MIS (Querleu-Morrow classification)
2. Exclusion criteria
  ① Any histological type other than adenocarcinoma, squamous cell carcinoma, or adenosquamous carcinoma of the uterine cervix.
  ② Tumour size greater than 4 cm.
  ③ Stage IA, II to IV disease according to the revised 2009 FIGO staging system.
  ④ Evidence of metastatic disease by conventional imaging studies; enlarged pelvic or aortic lymph nodes greater than 2 cm, or histologically positive lymph nodes.
  ⑤ A history of pelvic or abdominal radiotherapy.
  ⑥ Pregnancy.
  ⑦ Contraindication of surgery such as serious concomitant systemic disorders incompatible with the study to be decided at the discretion of the investigator.
  ⑧ Agreement to intra-operative lymphatic mapping without known allergies to triphenylmethane compounds, history of retroperitoneal surgery or pelvic irradiation, or cold knife or loop electrosurgical excision procedure cone biopsy within 4 weeks of enrollment.

### Primary endpoint

The primary endpoint of this study is the 4.5-year disease-free survival (DFS) rate, which is defined as the probability of no evidence of disease recurrence from the date of operation to the postoperative date of 4.5 years.

### Secondary endpoints

The secondary endpoints include the 4.5-year overall survival (OS) rate, recurrence pattern, and safety. The OS rate of 4.5 years is defined as the probability of survival from the date of operation to the postoperative date of 4.5 years. The recurrence pattern is evaluated by the location of recurrence in patients with confirmation of recurrence. Safety will be assessed by the rate of intraoperative organ injury, early (within 4 weeks after surgery), and late (from 4 weeks to 6 months after surgery) complications graded by Memorial Sloan Kettering Cancer Center (MSKCC) grading criteria [15]. Also, a detailed pathologic report, estimated blood loss, operation time, transfusion, and hospitalisation days would also be evaluated.

### Sample size

Based on our previous study, the 4.5-year DFS rate of patients with stage IB1 disease who underwent the abdominal radical hysterectomy was 90.9% [16], and the non-inferiority margin was 7.2% for MIS in the LACC trial [3]. Based on these results, we hypothesised that the 4.5-year DFS rate of patients who would undergo MIS using an endoscopic stapler would be the same value of 90.9%, and if the DFS does not differ by more than 7.2%, abdominal radical hysterectomy and MIS using an endoscopic stapler would be equivalent to each other. Therefore, the null hypothesis (H0) for this study is that the 4.5-year DFS rate of patients who would undergo MIS using an endoscopic stapler is 83.7%, whereas the alternative hypothesis (H1) is that the 4.5-year DFS rate of the patients is 90.9%. When we consider a three-year accrual and 4.5-year follow-up, at least a total of 13 events must happen, requiring a total of 111 patients assuming a statistical power of 80% and the one-tailed test of 5% significance. A total of 124 patients is needed, considering a drop-out rate of 10%.

### Patient discontinuation from the study

1. Patient withdrawal of consent.
2. If it is judged that it is difficult to participate in this clinical trial due to the health of the patient.
3. In case major clinical trial protocol violation or non-compliance is confirmed according to the evaluation of the principal investigator.
4. There is no patient discontinuation based on the biopsy results after surgery (i.e. positive resection margin, positive metastatic lymph node, positive cytology etc.)

### Statistical methods

The efficacy analysis will be performed in both per-protocol and intention-to-treat populations. Kaplan-Meier analysis will be done to estimate DFS and OS. The Cox proportional hazard model will be applied to estimate the hazard ratios and 95% confidence intervals. Although subjects were recruited according to the 2009 FIGO stage, the patients will be reclassified by the 2018 FIGO stage using the preoperative tumour size, and subgroup analysis will be performed.

## Discussion

The SOLUTION trial focused on using an endoscopic stapler to prevent tumor spillage, considered the leading risk factor for poor prognosis in patients with early-stage cervical cancer treated with minimally invasive radical hysterectomy [3, 4]. Recently updated guidelines recommend that minimally invasive radical hysterectomy should not be preferred and that surgeons should conduct sufficient counseling with the patient before radical hysterectomy [5, 6]. A large-scale retrospective study reported that extracorporeal colpotomy during MIS showed the comparable efficacy to abdominal radical hysterectomy in the LACC trial (3-year DFS rate, 96.8% vs 97.1%; 3-year OS rate, 98.5% vs 99%) [13], suggesting the preventive effect of extracorporeal colpotomy on tumour spillage.

Tumour size can increase the risk of tumour spillage during intracorporeal colpotomy. Recent studies demonstrated that patients with less than 2 cm tumours showed no difference in survival between abdominal radical hysterectomy and MIS [16, 17]. In terms of eligibility criteria, we only included IB1 disease and excluded IA and IB2 disease. In the LACC trial, there was no evidence for a difference in survival according to the surgical approach in IB2 patients because patients with a tumour size of 4 cm or more were excluded from the study. Therefore, in our study, IB2 patients with tumour sizes greater than 4 cm were excluded. For patients less than 2 cm in diameter, as shown in several studies, there was no difference in survival between the two surgical methods, so patients with IA disease were excluded in the SOLUTION trial. Moreover, cervical conisation before radical hysterectomy showed a protective effect on disease recurrence [17, 18]. These findings suggest that the risk of tumour spillage during MIS increases as tumour size increases, which requires specific methods to prevent tumour spillage, such as the use of an endoscopic stapler in this trial. Even though the no-look, no-touch technique which encased the tumour within the upper vaginal cuff prevented tumour spillage with a similar survival benefit to adnominal radical hysterectomy [12], this technique can be conducted by only surgeons proficient in vaginal surgery and may not be feasible for women with the narrow vaginal orifice.

On the other hand, the effect of using a uterine manipulator on disease recurrence and survival is still controversial. The Surgery in Cervical Cancer, Observational, Retrospective (SUCCOR) study reported that no use of a uterine manipulator and the use of manoeuvres to avoid tumour spread at the time of colpotomy during MIS was related with comparable outcomes to open surgery [7]. In contrast, the other study showed that using a uterine manipulator was not a factor associated with disease recurrence and survival [19]. In the latter study, earlier stage patients including IA1-IB1 were enrolled, whereas the former included only IB1 patients. Therefore, the use of intrauterine manipulators in IB1 patients is considered to be associated with a higher risk of relapse, but there appears to be insufficient evidence of risk. So, we recommended that participating researchers will use a vaginal tube that does not penetrate through the cervical tumour. Even if they prefer a uterine elevator that requires a tip inserted through the cervical tumour, bilateral tubal ligation is required before the main surgical procedure to minimise the risk of tumour dissemination by using a uterine manipulator in this trial.

Furthermore, the inexperience of surgeons in using an endoscopic stapler during MIS can act as a bias in this trial. In the LACC trial, surgeons should have provided evidence of a minimal number of ten documented laparoscopic radical hysterectomies conducted and the outcomes of these operations with a video recording of at least one laparoscopic radical hysterectomy before they participated [3]. An ongoing Chinese phase III trial comparing the efficacy between abdominal radical hysterectomy and MIS requires at least 100 unselected, consecutive radical hysterectomy cases of investigators to qualify their skills and learning curves [20]. In the SOLUTION trial, TMC will verify the surgical experience of the participating investigators and the proficiency of using an endoscopic stapler to a similar extent to the two relevant trials. Furthermore, vaginal opening and vaginal closure after colpotomy are performed in the same way as laparoscopic hysterectomy; the risk of complications related to the surgical procedure is not expected to increase.

In terms of lymphadenectomy during SOLUTION trial, the investigators agreed to recommend systematic pelvic lymphadenectomy but permitted sentinel lymph node mapping during the surgery. First, SENTICOL II trial showed that sentinel lymph node mapping alone was not associated with worse outcomes than sentinel lymph node mapping with pelvic lymph node dissection [21]. Second, LACC trial protocol also allowed the operators to perform sentinel lymph node mapping and assessed the feasibility of sentinel lymph node biopsy [3]. These two studies support allowing sentinel lymph node mapping alone during the trial progression.

Currently, robot-assisted approach to cervical cancer (RACC) trial is recruiting early-stage cervical cancer (NCT03719547) [22]. This randomised non-inferiority trial protocol prohibits using uterine manipulators and recommends closing the vagina before colpotomy, but not mandatory. Although the RACC trial makes an effort to minimise tumour spillage in common with the SOLUTION trial, it compares the oncologic outcome of robot-assisted surgery to open surgery. On the other hand, SOLUTION trial is a phase II trial to evaluate the oncologic outcome of endoscopic vaginal stapling, either conventional laparoscopic or robotic. A randomised controlled trial comparing laparoscopic surgery with endoscopic stapling and laparotomy is needed based on the results of this study in the future.

The limitations of this study are as follows: first, the unexpected metastatic lesion may affect the washing cytology result. This should be interpreted with caution in conjunction with pathologic outcomes. Second, sentinel lymph node mapping and para-aortic lymphadenectomy were permitted and performed at the surgeon” discretion. Up to now, limited Evidence supports performing surgical staging for early-stage cervical cancer; still, there are chances to detect small volume metastasis, the prognostic role is not well elucidated. Therefore, each investigator will decide whether to perform para-aortic lymphadenectomy or not according to the patient status, such as large tumour volume, elevated tumour markers, etc. Third, 4.5-year DFS instead of 5-year DFS is used for the primary endpoint, not conventionally used as a trial endpoint. However, using a 4.5-year DFS, we can attain a primary endpoint with minimal sample size, follow-up period, and comparable results with LACC trial.

To summarise, we expect that intracorporeal colpotomy using an endoscopic stapler may prevent tumour spillage during minimally invasive radical hysterectomy for stage IB1 disease. Moreover, we will perform the peritoneal washing cytology three times during MIS as follows: before insertion of a uterine manipulator; before intracorporeal colpotomy after resecting the vagina using the endoscopic stapler; after the closure of the vaginal stump. Preoperative cytology will be able to evaluate the presence of microscopic tumors in the abdominal cavity before surgery. We also expect to detect tumour spillage through consecutive peritoneal washing cytology and will evaluate how the detection of tumour spillage would affect the prognosis and recurrence pattern in this trial.

## Data Availability

The datasets used and/or analysed during the current study are available from the corresponding author (Hee Seung Kim) at reasonable request.

## Abbreviations

CCRT: Concurrent chemoradiotherapy
DFS: Disease-free survival
FIGO: The International Federation of Gynecology and Obstetrics
LACC: Laparoscopic Approach to Cervical Cancer
MIS: Minimally invasive surgery
MSKCC: Memorial Sloan Kettering Cancer Center
OS: Overall survival
SOLUTION: Safety Of Laparoscopic or robotic radical surgery Using an endoscopicsTapler for Inhibiting tumOr spillage of cervical Neoplasms
SUCCOR: SUrgery in Cervical Cancer, Observational, Retrospective
TMC: Trial Monitoring Committee
RACC: Robot-assisted approach to cervical cancer
